# Association Between Cribriform Architecture and Lymphovascular Invasion in Prostate Cancer

**DOI:** 10.3390/jcm15031032

**Published:** 2026-01-28

**Authors:** Jacqueline Chan, Yetkin Tuac, Okan Argun, Christina M. Breneman, Nora Seeley, Haley N. Moriarty, Keerthana Senthil Kumar, Fallon E. Chipidza, Jonathan E. Leeman, Mutlay Sayan

**Affiliations:** 1 Department of Radiation Oncology, Brigham and Women’s Hospital and Dana Farber Cancer Institute, Harvard Medical School, Boston, MA 02115, USA; 2 Department of Statistics, Ankara University, Ankara 06100, Türkiye

**Keywords:** prostate cancer, cribriform architecture, lymphovascular invasion, micrometastatic disease

## Abstract

**Background/Objectives:** Cribriform architecture is an adverse histopathologic feature in prostate cancer and has been associated with poor oncologic outcomes. Emerging evidence suggests that cribriform-positive tumors may behave as a biologically non-localized disease, raising the possibility of early occult dissemination. Lymphovascular invasion (LVI) is a key pathological marker of metastatic potential, but its relationship with cribriform architecture has not been evaluated. We examined the association between cribriform morphology and LVI to provide biological context for the aggressive clinical course of cribriform-positive prostate cancer. **Methods:** We performed a retrospective analysis of patients with prostate adenocarcinoma who underwent radical prostatectomy and had available clinicopathologic data. Cribriform architecture was determined by a centralized pathology review, and LVI status was obtained from original pathology reports. Unadjusted associations were evaluated using contingency tables. Multivariable logistic regression was used to assess whether cribriform architecture was independently associated with LVI after adjustments for Gleason score, tumor stage, and nodal status. **Results:** Among 338 patients, 28 (8.3%) had LVI and 123 (36.4%) had cribriform architecture. LVI was more common in cribriform-positive than cribriform-negative tumors (17.9% vs. 2.8%; *p* < 0.001), corresponding to a crude odds ratio (OR) of 7.6 (95% CI, 3.0–19.3). Cribriform architecture remained independently associated with LVI after adjustment (adjusted OR, 5.20; 95% CI, 2.12–1.40; *p* < 0.001). **Conclusions:** Cribriform architecture is strongly and independently associated with LVI, supporting a biological link between cribriform morphology and early metastatic dissemination. These findings support the design of prospective, biomarker-driven studies to evaluate treatment intensification strategies in this high-risk subgroup.

## 1. Introduction

The accurate detection of biologically aggressive disease among patients with localized prostate cancer remains a significant clinical challenge. Although the conventional risk stratification relies on clinical stage, prostate-specific antigen (PSA) level, and Gleason score, substantial heterogeneity in long-term outcomes persists among patients treated with curative intent [1,2,3,4]. Cribriform architecture has emerged as a histopathologic feature of interest, following its uniform classification as Gleason pattern 4 and its consistent association with adverse oncologic outcomes [5,6,7,8,9,10,11,12,13,14,15,16]. In a recent secondary analysis of the PROTECT randomized clinical trial, cribriform-positive tumors carried a markedly higher long-term risk of metastatic progression; notably, among these patients, radiotherapy combined with neoadjuvant androgen deprivation therapy (ADT) was associated with a lower risk of metastasis than surgery [16]. These findings raise the possibility that cribriform morphology identifies tumors with a propensity for early, clinically occult dissemination that may be more effectively addressed with systemic therapy in addition to local treatment.

While the adverse prognostic implications of cribriform morphology are now well recognized, the biological mechanisms underlying its aggressive clinical behavior remain incompletely defined. One plausible explanation is an increased propensity for vascular or lymphatic invasion. Lymphovascular invasion (LVI) is a well-established adverse pathological feature in prostate cancer and is associated with higher Gleason grade, advanced pathologic stage, lymph node metastasis, and poor oncologic outcomes [17,18,19,20,21]. As a pivotal step in the metastatic cascade, LVI enables dissemination to regional lymph nodes and distant organs, and its detection may have implications for treatment intensification strategies [22,23,24,25].

However, the relationship between cribriform morphology and LVI has not been systematically evaluated. This gap is particularly relevant given the emerging evidence suggesting that cribriform-positive tumors may behave as a biologically non-localized disease at diagnosis. Consequently, we examined whether cribriform morphology is associated with pathological features reflecting early metastatic potential, specifically LVI, in a contemporary cohort of patients undergoing radical prostatectomy, thereby providing biological context for its adverse prognostic significance and potential implications for treatment selection.

## 2. Materials and Methods

### 2.1. Study Population

**Figure 1:**
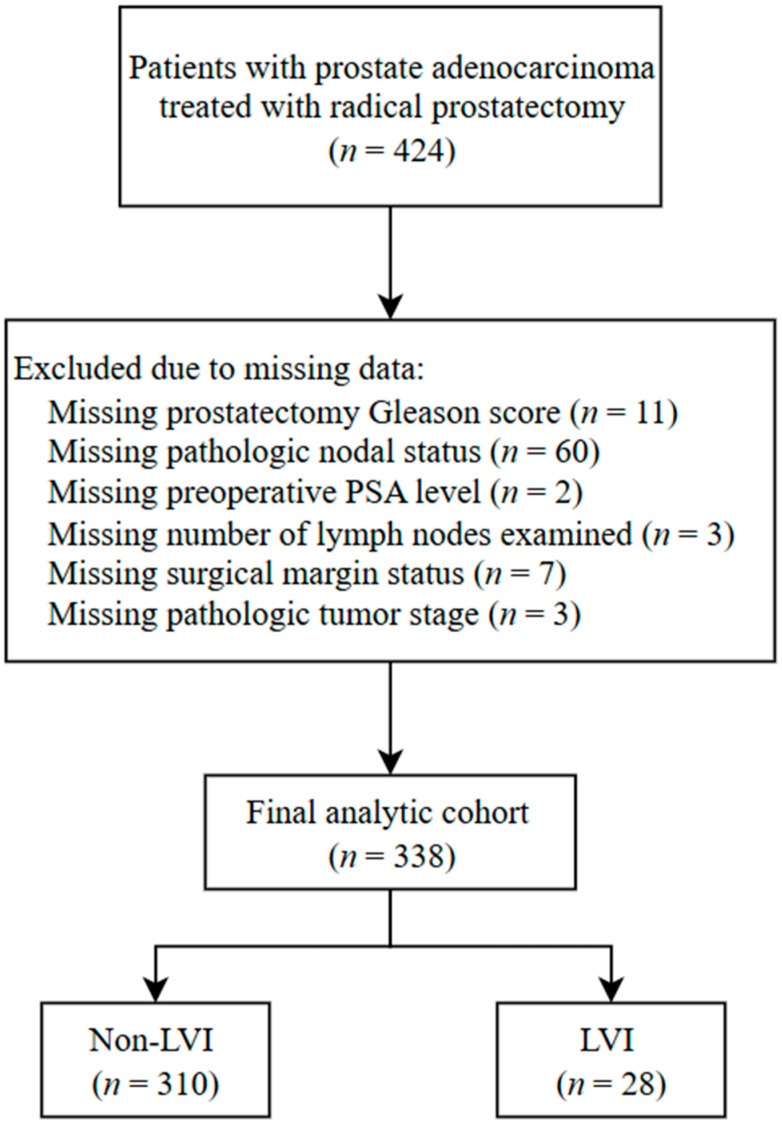
Flow diagram illustrating patient selection for the study. Abbreviations: LVI, lymphovascular invasion; PSA, prostate-specific antigen.

This retrospective study analyzed patients with prostate adenocarcinoma from The Cancer Genome Atlas (TCGA) prostate cancer cohort. Patients were eligible for inclusion if they underwent radical prostatectomy between January 2000 and December 2023 and had available clinicopathologic data, including assessment of cribriform architecture and LVI. Patients were excluded if they were missing any of the following variables required for analysis: prostatectomy Gleason score, pathologic tumor stage, nodal status, preoperative prostate-specific antigen level, prostatectomy margin status, or number of lymph nodes examined. Patient selection and exclusions due to missing data are detailed in Figure 1. No institutional or locally accrued patient data were included. Cribriform architecture was determined based on a centralized pathology review of digitized radical prostatectomy slides performed as part of the TCGA pathology review process. As previously described [5], this review was conducted by a team of three pathologists with expertise in genitourinary pathology, with the primary objective of identifying the presence of cribriform architecture using established morphologic criteria [26,27]. The distinction between cribriform pattern and intraductal carcinoma was based on accepted histopathologic features described in prior literature. While the review followed a consistent histopathologic approach, information regarding reviewer blinding and formal interobserver reproducibility was not available, reflecting a limitation of this retrospective analysis. LVI status was obtained from the original pathology reports available within the TCGA dataset and recorded as a binary variable (present vs. absent).

This study was conducted in accordance with the Declaration of Helsinki. An institutional review board waiver was obtained from the Dana-Farber Cancer Institute for the use of de-identified administrative and pathology data, and the requirement for informed consent was waived.

### 2.2. Statistical Methods

#### 2.2.1. Comparison of Clinical and Pathologic Factors Stratified by LVI

This study followed a prespecified statistical analysis plan. Descriptive statistics were used to summarize the clinical and pathological characteristics of 338 patients, stratified by the presence or absence of LVI. Categorical variables, including cribriform pattern, prostatectomy margin status, nodal status, tumor stage, and Gleason group, were compared using Pearson’s chi-squared test or Fisher’s exact test [28]. Continuous variables, such as age, baseline PSA, and number of lymph nodes examined, were evaluated using the Wilcoxon rank-sum test due to non-normal distributions [29].

Unadjusted associations between cribriform pattern and LVI were evaluated using 2 × 2 contingency tables. Incidence proportions of LVI among cribriform-positive versus cribriform-negative tumors were used to calculate unadjusted risk ratios (RRs), odds ratios (ORs), and corresponding 95% confidence intervals (CIs). Statistical significance for unadjusted associations was assessed using chi-squared or Fisher’s exact tests, as appropriate.

#### 2.2.2. Covariate-Adjusted Odds Ratios for LVI

The primary objective was to determine whether cribriform architecture was independently associated with LVI. Multivariable logistic regression analysis was performed to adjust for potential confounders, including prostatectomy Gleason score (≤7 vs. 8–10), pathologic tumor stage (T2, T3, and T4), and prostatectomy nodal status (N0 vs. N1) [30]. In addition to adjusted modeling, crude associations between cribriform pattern and LVI were evaluated using cohort-based incidence risk ratios derived from 2 × 2 contingency tables [31]. Model discrimination was subsequently assessed using receiver operating characteristic (ROC) curve analysis [32]. Adjusted odds ratios (aORs) with 95% CIs were reported for all predictors. Potential effect modification between cribriform pattern and Gleason group was explored by including an interaction term.

The multivariable logistic regression model included four pre-specified predictors. These variables were selected a priori based on established biological relevance and prior literature, rather than through data-driven or automated variable selection procedures. Given the number of LVI events (*n* = 28), the resulting events-per-variable (EPV) ratio was approximately 7. To minimize the risk of overfitting, all covariates were pre-specified based on strong clinical relevance rather than data-driven selection procedures. Model discrimination was evaluated using ROC curve analysis. Firth penalized logistic regression [33] was performed to assess the robustness of the observed associations. Because the number of LVI events was limited, the primary model was determined to be parsimonious and included four covariates with the strongest biological rationale. Pre-RP PSA, margin status, and adjuvant treatment were evaluated as potential confounders and were included in sensitivity analyses to assess robustness of the cribriform–LVI association.

All statistical tests were two-sided, with statistical significance defined as *p* < 0.05. All analyses were conducted using R software (version 4.2.3).

## 3. Results

### 3.1. Comparison of the Clinical and Pathologic Factors Stratified by LVI

Among the 338 patients included in this study, 28 (8.28%) had LVI, and 123 (36.39%) had cribriform architecture. The distribution of clinical and pathological characteristics stratified by LVI status is shown in Table 1. Compared with patients without LVI, those with LVI had higher pre-radical prostatectomy PSA levels (median, 11 ng/mL [IQR, 8–26] vs. 8 ng/mL [IQR, 5–11]; *p* < 0.001), were more likely to have high-grade disease (Gleason score 8–10: 75% vs. 40%; *p* < 0.001), had more advanced pathologic tumor stage (T3: 82% vs. 63%; T4: 11% vs. 1%; *p* < 0.001), and more likely to have pathologic lymph node involvement (N1: 36% vs. 14%; *p* = 0.005).

The prevalence of cribriform architecture was substantially higher among patients with LVI compared with those without LVI (79% vs. 33%; *p* < 0.001), indicating a strong unadjusted association between cribriform morphology and LVI. Using cohort-based estimates derived from a 2 × 2 contingency table, LVI was present in 22 of 123 cribriform-positive tumors (17.9%) compared with 6 of 215 cribriform-negative tumors (2.8%). This corresponded to a crude risk ratio of 6.4 and an OR of 7.6 (95% CI, 3.0–19.3; *p* < 0.001), demonstrating a markedly increased likelihood of LVI among tumors with cribriform architecture.

### 3.2. Covariate-Adjusted Odds Ratios for LVI

As shown in Table 2, the presence of cribriform architecture remained independently associated with LVI after adjustment for prostatectomy Gleason score, pathologic tumor stage, and nodal status (adjusted OR, 5.20; 95% CI, 2.12–14.40; *p* < 0.001).

In sensitivity analyses, additionally adjusting for pre-RP PSA, margin status, and adjuvant treatment, the association between cribriform pattern and LVI remained qualitatively unchanged.

**Figure 2:**
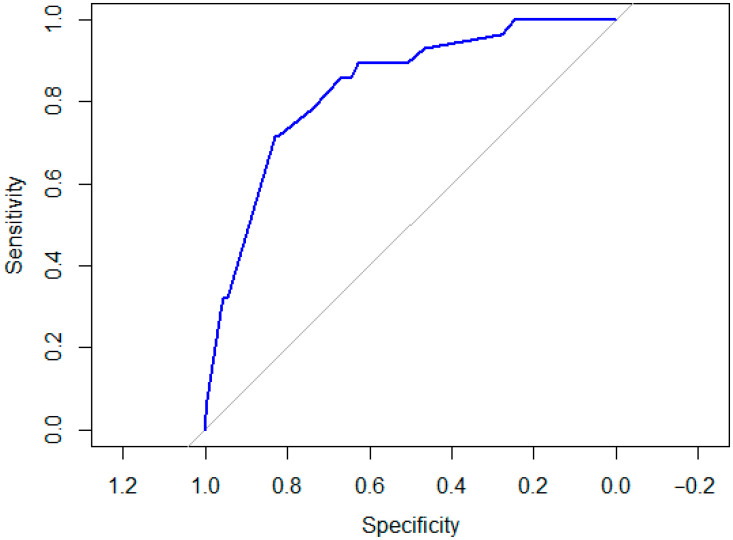
Receiver operating characteristic (ROC) curve for predicting lymphovascular invasion.

The multivariable logistic regression model demonstrated good discrimination for LVI, with an area under the ROC curve of 0.83 (Figure 2). This indicates that the model correctly distinguishes LVI-positive from LVI-negative patients in approximately 83% of pairwise comparisons. The ROC curve showed clear separation from the diagonal reference line, indicating consistent performance across a range of sensitivity and specificity thresholds.

Collectively, these findings indicate that the variables included in the model—cribriform pattern, prostatectomy Gleason score, pathologic tumor stage, and nodal status—jointly contribute to discrimination of LVI status within this cohort. The observed model performance supports the robustness of the association between cribriform architecture and LVI in multivariable analysis. In sensitivity analyses additionally adjusting for pre-RP PSA, margin status, and adjuvant treatment, the association between cribriform pattern and LVI remained unchanged, and inclusion of these variables did not materially improve model fit or alter effect estimates. There was no evidence of effect modification between cribriform architecture and Gleason group on the risk of LVI (interaction aOR, 1.88 95% CI, −1.34–2.67; *p* = 0.527), and therefore interaction terms were not retained in the final model.

Perineural invasion (PNI) is a well-established adverse pathologic feature that can be identified on both biopsy and prostatectomy specimens. Although PNI was not independently associated with LVI after multivariable adjustment in our cohort (aOR, 0.87 95% CI, 0.34–2.49; *p* = 0.783), its co-occurrence with cribriform and/or LVI may still reflect a biologically aggressive phenotype.

## 4. Discussion

In this analysis of patients undergoing radical prostatectomy, we examined the association between cribriform architecture and LVI, a key pathological marker of early metastatic potential. We found that cribriform morphology was strongly associated with LVI, with a nearly eightfold higher unadjusted odds and a persistently elevated risk after adjustment for established adverse pathological features. Importantly, the association was further strengthened by the favorable discriminatory performance of the multivariable model, highlighting the robustness of the observed relationship. The clinical significance of these findings lies in providing biological context for the consistently adverse outcomes observed in cribriform-positive prostate cancer and supporting the hypothesis that cribriform architecture identifies tumors with an increased propensity for early vascular or lymphatic dissemination—an insight with direct implications for postoperative risk stratification and treatment selection.

A growing body of evidence suggests that adverse outcomes following definitive local therapy for prostate cancer may partly reflect the presence of micrometastatic disease at the time of diagnosis. Micrometastatic disease refers to tumor cell dissemination beyond the primary prostate tumor that remains below the detection threshold of conventional imaging and routine histopathologic assessment [34]. Multiple studies using immunohistochemical and molecular techniques have demonstrated that occult tumor cells can be identified in pelvic lymph nodes, bone marrow, or peripheral blood of patients with clinically localized disease, and their presence is associated with higher rates of biochemical recurrence following radical prostatectomy [35,36,37,38]. These observations indicate that a subset of patients classified as having localized prostate cancer may already harbor biologically systemic disease at presentation.

Within this framework, our findings suggest that LVI may represent a key mechanistic link between cribriform architecture and early metastatic dissemination. LVI reflects the intravasation of tumor cells into lymphatic or blood vessels and is widely regarded as a critical step in the metastatic cascade. The strong and independent association observed between cribriform morphology and LVI in our study supports the hypothesis that cribriform-positive tumors possess an enhanced capacity for vascular or lymphatic spread, thereby facilitating micrometastatic seeding before definitive local therapy. This biological interpretation is consistent with prior molecular studies demonstrating that micrometastatic tumor cells share clinicopathologic and prognostic features with overt nodal metastases, and that their presence is independently associated with disease recurrence even in patients without histologically detectable lymph node involvement [34,35,36].

Importantly, advances in sensitive detection modalities, including prostate-specific membrane antigen (PSMA)–based imaging, have further refined the concept of micrometastatic disease by revealing sites of tumor spread previously considered occult [39,40,41]. Although advances in imaging have improved the detection of small-volume metastatic disease, pathological evidence of LVI reflects an earlier step in the metastatic process. Taken together, these data support a model in which cribriform architecture identifies a biologically aggressive subset of prostate cancer characterized by early vascular or lymphatic invasion and an increased likelihood of micrometastatic disease at diagnosis.

These biological insights may help contextualize recent findings from randomized data suggesting differential treatment effects in patients with cribriform-positive prostate cancer. In a secondary analysis of the PROTECT trial, cribriform architecture was associated with a substantially increased long-term risk of metastatic progression, and this risk appeared to be mitigated by radiotherapy combined with neoadjuvant androgen deprivation therapy, but not by surgery alone [16]. Although treatment assignment in PROTECT was not stratified by cribriform status and causal inferences should be made cautiously, the observed benefit of systemic therapy in this subgroup is biologically consistent with a disease phenotype characterized by early dissemination. In this context, our finding that cribriform morphology is strongly associated with LVI provides pathological support for the hypothesis that cribriform-positive tumors may be associated with pathological features consistent with early metastatic potential—disease that is unlikely to be adequately addressed by local therapy alone but may be more effectively treated with the addition of systemic therapy.

From a clinical perspective, these findings suggest that histopathologic features such as cribriform architecture, particularly when accompanied by LVI, may help refine biological risk stratification beyond conventional clinicopathologic variables. The immediate application of these findings should prioritize guiding the design of future prospective studies. Specifically, identification of cribriform-positive disease with associated LVI may help identify a biologically distinct subset of patients who could be considered for inclusion in randomized clinical trials evaluating treatment intensification strategies following radical prostatectomy. Incorporating these pathological features into trial eligibility criteria or stratification schemas may enable more precise evaluation of whether additional systemic or multimodality therapy is necessary to improve long-term outcomes in this population. If validated in prospective trials, these histopathologic features could ultimately support more informed and biologically driven treatment selection following radical prostatectomy.

In addition to postoperative pathology, consideration of these features earlier in the disease course may also be relevant. While LVI may be reported on biopsy specimens, assessment of cribriform architecture at diagnosis is variable and not uniformly incorporated into routine biopsy reporting. Evaluation of these features at the biopsy stage could enhance early biological risk stratification and inform initial treatment discussions. However, biopsy pathology reports and specimens were not available in the present dataset, precluding direct assessment in this study. Future prospective studies evaluating these features on diagnostic biopsy specimens are warranted.

In addition to vascular and lymphatic pathways, PNI represents another adverse histopathologic feature [42,43,44] that may coexist with cribriform architecture and LVI. Although PNI was not independently associated with LVI after multivariable adjustment in our cohort, its presence alongside cribriform morphology and/or LVI may still reflect a biologically aggressive tumor phenotype. Rather than acting as isolated risk factors, these features may capture complementary pathways of tumor spread, including perineural, lymphatic, and vascular dissemination. Consideration of such combined adverse pathologic features may therefore enhance biological risk stratification and inform the design of future studies aimed at identifying patients at increased risk for early disease dissemination.

Despite these insights, this study has several limitations that warrant consideration. First, its retrospective design introduces the potential for selection bias and limits causal inference. Second, LVI status was derived from routine pathology reports rather than centralized slide re-review, which may introduce variability in assessment but reflects real-world pathology practice and enhances the generalizability of our findings. Furthermore, information regarding the use of immunohistochemical stains for LVI (e.g., D2-40 or CD31/CD34) was not available, and LVI was recorded based on routine pathology reports, which may introduce variability but reflects real-world practice. Third, the TCGA cohort represents a surgically treated population enriched for higher-risk disease, which may limit generalizability to patients managed with primary radiation therapy or active surveillance. Finally, while our findings support a biologically plausible link between cribriform architecture and early metastatic potential, prospective studies with standardized pathological assessment and long-term clinical follow-up are required to validate these observations.

## 5. Conclusions

This study demonstrates a strong association between cribriform architecture and LVI, providing biological context for the aggressive clinical behavior consistently observed in cribriform-positive prostate cancer. These findings support the hypothesis that cribriform morphology identifies tumors with an increased propensity for early metastatic dissemination, emphasizing the need for prospective, biomarker-driven studies to determine whether treatment intensification strategies may improve outcomes in this biologically high-risk subgroup.

## Figures and Tables

**Table 1:** Baseline clinical and pathologic factors stratified by LVI.

	Non-LVI (*n* = 310)	LVI (*n* = 28)	*p*
Age (years), median (IQR)	62 (56, 66)	62 (57, 65)	0.700
Pre-RP PSA, ng/mL, median (IQR)	8 (5, 11)	11 (8, 26)	<0.001
Prostatectomy Gleason score, No. (%)			<0.001
≤7	185 (60%)	7 (25%)	
8–10	125 (40%)	21 (75%)	
Prostatectomy tumor stage, No. (%)			<0.001
T2	111 (36%)	2 (7.1%)	
T3	196 (63%)	23 (82%)	
T4	3 (1.0%)	3 (11%)	
Prostatectomy margin status, No. (%)			0.300
Negative	210 (68%)	16 (57%)	
Positive	100 (32%)	12 (43%)	
Adjuvant treatment, No. (%)	37 (12%)	8 (29%)	
Number of lymph nodes examined, median (IQR)	9 (5, 15)	13 (6, 19)	0.057
Prostatectomy nodal status, No. (%)			0.005
N0	267 (86%)	18 (64%)	
N1	43 (14%)	10 (36%)	
PNI, No. (%)			0.900
No	70 (23%)	6 (21%)	
Yes	240 (77%)	22 (79%)	
Cribriform Pattern, No. (%)			<0.001
No	209 (67%)	6 (21%)	
Yes	101 (33%)	22 (79%)	

Abbreviations: LVI, Lymphovascular invasion; RP, radical prostatectomy; PSA, prostate-specific antigen; PNI, perineural invasion; IQR, interquartile range.

**Table 2:** Multivariable Firth penalized logistic regression analysis for predictors of LVI.

	aOR	95% CI	*p*
Cribriform Pattern			
No	Reference	Reference	
Yes	5.20	2.12–14.40	<0.001
Prostatectomy Gleason score			
≤7	Reference	Reference	
8–10	2.08	0.81–5.93	0.13
Prostatectomy tumor stage			
T2	Reference	Reference	
T3	2.55	0.74–13.3	0.15
T4	33.6	3.81–365	0.002
Prostatectomy nodal status			
N0	Reference	Reference	
N1	2.31	0.91–5.76	0.078

Abbreviations: LVI, lymphovascular invasion; CI, confidence interval; aOR, adjusted odds ratio.

## Data Availability

The original contributions presented in this study are included in the article. Further inquiries can be directed to the corresponding author.

## Abbreviations

The following abbreviations are used in this manuscript:

ADT	Androgen deprivation therapy
AUC	Area under the curve
CI	Confidence interval
EPV	Events-per-variable
LVI	Lymphovascular invasion
OR	Odds ratio
PET	Positron emission tomography
PNI	Perineural invasion
PSA	Prostate-specific antigen
PSMA	Prostate-specific membrane antigen
ROC	Receiver operating characteristic
RP	Radical prostatectomy
TCGA	The Cancer Genome Atlas
